# A Novel Transcriptional Factor *Nkapl* Is a Germ Cell-Specific Suppressor of Notch Signaling and Is Indispensable for Spermatogenesis

**DOI:** 10.1371/journal.pone.0124293

**Published:** 2015-04-14

**Authors:** Hidenobu Okuda, Hiroshi Kiuchi, Tetsuya Takao, Yasushi Miyagawa, Akira Tsujimura, Norio Nonomura, Haruhiko Miyata, Masaru Okabe, Masahito Ikawa, Yoshitaka Kawakami, Naoki Goshima, Morimasa Wada, Hiromitsu Tanaka

**Affiliations:** 1 Department of Urology, Osaka University Graduate School of Medicine, Osaka, Japan; 2 Department of Urology, Juntendo University Hospital, Tokyo, Japan; 3 Animal Resource Center for Infectious Diseases, Research Institute for Microbial Diseases, Osaka University, Osaka, Japan; 4 National Institute of Advanced Industrial Science and Technology, Tokyo, Japan; 5 Molecular Biology Division, Faculty of Pharmaceutical Sciences, Nagasaki International University, Nagasaki, Japan; University Hospital of Münster, GERMANY

## Abstract

Spermatogenesis is an elaborately regulated system dedicated to the continuous production of spermatozoa via the genesis of spermatogonia. In this process, a variety of genes are expressed that are relevant to the differentiation of germ cells at each stage. Although Notch signaling plays a critical role in germ cell development in *Drosophila* and *Caenorhabditis elegans*, its function and importance for spermatogenesis in mammals is controversial. We report that *Nkapl* is a novel germ cell-specific transcriptional suppressor in Notch signaling. It is also associated with several molecules of the Notch corepressor complex such as CIR, HDAC3, and CSL. It was expressed robustly in spermatogonia and early spermatocytes after the age of 3 weeks. *Nkapl*-deleted mice showed complete arrest at the level of pachytene spermatocytes. In addition, apoptosis was observed in this cell type. Overexpression of NKAPL in germline stem cells demonstrated that *Nkapl* induced changes in spermatogonial stem cell (SSC) markers and the reduction of differentiation factors through the Notch signaling pathway, whereas testes with *Nkapl* deleted showed inverse changes in those markers and factors. Therefore, *Nkapl* is indispensable because aberrantly elevated Notch signaling has negative effects on spermatogenesis, affecting SSC maintenance and differentiation factors. Notch signaling should be properly regulated through the transcriptional factor *Nkapl*.

## Introduction

Spermatogenesis is a highly specialized and complex process. Spermatogonia proliferate, and some differentiate into spermatocytes, which undergo meiosis to yield haploid spermatids. Finally, spermatids give rise to spermatozoa with dramatic morphological changes. During this process, a variety of developmental stage-specific molecules and transcriptional factors are elaborately orchestrated and support spermatogenesis. Abnormalities in these factors have been considered a cause of male factor infertility, and the disruption of these indispensable genes for spermatogenesis leads to spermatogonial stem cell (SSC) depletion or the arrest of maturation in mouse models [1–3]. Therefore, discovery of novel genes and transcriptional factors associated with germ cell differentiation is essential to understanding the mechanism of spermatogenesis and the etiology of male factor infertility, although these remain to be well elucidated.

Previously, we reported germ cell-specific intronless genes that have functional enzymatic activity for energy metabolisms [4,5]. These genes were assumed to originate from the parental genes by the process of retrotransposition, which has contributed to the evolution of species by generating novel functional genes in spite of creating pseudogenes [6]. These genes are often expressed specifically in germ cells and are associated with male infertility [7].

*Nkapl (*NFkB activating protein-like) has several characteristic features of a retrotransposition gene from *Nkap* (NFkB activating protein), with intronlessness and high identity (67% in mice and 70% in humans), and is conserved from primitives to humans. *NKAP* was identified as a RIP (receptor-interacting protein), which can potentially activate NFkB as well as other RIPs [8]. It was then proven to be a transcriptional repressor in Notch signaling and essential for T cell development [9]. These findings suggested that *Nkapl* could play a critical role in spermatogenesis in adult testis as a transcriptional factor affecting the Notch signaling pathway, although this has never been examined.

Notch signaling is the highly evolutionary conserved pathway that is initiated in response mainly to five Notch ligands of the Delta-Serrate-Lag (DSL) type (Jag1 and Jag2 and delta-like 1 (Dll1), Dll3 and Dll4) [10]. In mammals, there are four kinds of Notch transmembrane receptors (NOTCH1-4) to Notch ligands [11]. On interacting ligands and receptors, Notch receptors cause proteolytic cleavage and subsequently produce NICD (Notch intracellular domain). NICD translocates to the nucleus and interacts with CSL (Cbf1/Rbp-jk) to initiate the transcription of Notch target genes such as *Hes1* and *Hes5*, orthologues of *Hes* (Hairy and enhancer of split) family genes [12]. In the absence of NICD, co-repressor complex, including CIR (Corepressor interacting with RBPJ) and HDAC (Histone deacetylase), suppresses the transcription of target genes by binding to CSL to regulate the transcription of downstream target genes. Notch signaling has important roles in the developmental process, determination of cell fate, and disease and is intimately regulated [13,14].

It has been reported that Notch signaling is critical for germ cell development in *Caenorhabditis elegans* [15], *Drosophila* [16,17], and *Xenopus* [18]. Notch signaling is also essential for Sertoli cells and Leydig cells in the fetal development of mammalian testis. Mutant mice with constitutively active Notch1 in Sertoli cells showed aberrant exit from mitotic arrest, migration, and differentiation before birth [19].Furthermore, constitutively active Notch1 signaling in gonadal somatic cells caused dramatic Leydig cell loss, suggesting its necessity for the maintenance of Leydig progenitor cells [20]. However, the function and necessity of Notch signaling in germ cells and spermatogenesis have not yet been examined well. Some articles reported that NOTCH1 was dispensable for normal spermatogenesis from phenotypic analyses of conditional *Notch1*-deleted mice [21–23], and Notch family receptors and their ligands were expressed stage-specifically in germ cell and testicular somatic cells [24–26]. Although there are some inconsistencies in regard to the localization of each Notch orthologue, spermatogonia are considered as one of the important expression sites of Notch family receptors, and NOTCH1 has some effects on germ cell development to some extent [26]. Furthermore, mice with germ cell-specific overexpression of NOTCH1 showed reduced spermatogenesis progressively affected by age [23]. These findings suggest that Notch signaling has some functional roles in both germ cells and spermatogenesis.

In this study, we will show that novel germ cell-specific gene *Nkapl* is a functional nuclear protein expressed robustly in differentiating spermatogonia and spermatocytes after puberty in mice and is a repressor of Notch signaling, interacting with co-repressor proteins and transcription factors. Furthermore, NKAPL affects transcription of SSC markers and differentiation through the Notch signaling pathway and is an indispensable gene for spermatogenesis.

## Materials and Methods

### RNA extraction

Total RNA was extracted from testes or cultured cells using Trizol (Life Technologies) according to the manufacturer’s protocols. To remove genomic DNA contamination, extracted RNA was treated with DNaseI recombinant (Roche).

### RT-PCR and qRT-PCR

The total RNA was reverse-transcribed into cDNA using the PrimeScript kit (TaKaRa) according to the described protocols. The samples without reverse-transcriptase were defined as the negative control. RT-PCR was performed using Gflex Tks (TaKaRa) according to the manufacturer’s protocol. All qRT-PCR were performed using SYBR Premix EX TaqTM (Tli RNaseH Plus) (TaKaRa). PCR products were quantified by the Thermal Cycler Dice Real Time System II. *Actb* was amplified at the same time as an internal control. All qRT-PCR results were normalized to *Actb* gene expression, and those of control samples were assumed to equal 1. Primer sets are shown in S3 Table. Independent PCR reactions for all samples were performed at least three times, and expression levels were normalized to those of the internal control *Actb*.

### Vector construction

Full-length human *NKAP* and *NKAPL* cDNA were gifts from N. Goshima. Full-length mouse *Nkap* and *Nkapl* were amplified by PCR from the testis-specific cDNA library. These fragments were inserted into pcDNA3.1+ with FLAG tag (DYKDDDDK) for synthesizing recombinant protein. pNFkB and pTK-RL were purchased from Agilent Technologies as reporter plasmids in the luciferase assay for analyzing NFkB activation. Full-length mouse *Cir*, *Hdac3*, *Csl*, and *NICD* of Notch1-3 were obtained by PCR from mouse testis cDNA and were inserted with FLAG tag into pcDNA3.1+. For analysis of the transcriptional assay, pEluc-CSLs were generated by inserting CSL binding consensus sequence (GTGGGAA×4) and SV40 promoter sequence into pEluc (Promega).

### Transient transfection experiments

293T cells were incubated and transfected with Lipofectamine 2000 (Invitrogen) according to the manufacturer’s protocol. After 24 hours of incubation, the 293T cells were washed with PBS and lysed and sonicated with RIPA buffer (Santa Cruz Biotechnology) or HEPES buffer including protease inhibitor cocktail (Sigma-Aldrich).

### Immunoprecipitation

Forty microliters of Dynabeads Protein G (Invitrogen) was reacted with 2.5 μl of GFP monoclonal antibody (WAKO) for 10 minutes. Cell lysates cotransfected with GFP tagged NKAPL and FLAG tagged CIR, HDAC3 or CSL were separated by centrifugation. Their supernatants were added to the magnetic beads conjugated with GFP antibody and incubated for 10 minutes at room temperature. Magnetic beads were collected and washed with PBS four times. Proteins were eluted with 25 μl of SDS-sample buffer.

### Western blotting

Eight micrograms of transfected whole-cell extracts or 8 μl of immunoprecipitated protein elution was separated by SDS-PAGE and electroblotted onto PVDF membranes. After blocking with 4% BlockAce (DS Pharma Biomedical) in PBS-T, the membranes were reacted with primary and secondary antibodies at room temperature for 1 hour. The primary and secondary antibodies are listed in S4 Table. The antigen-antibody complexes were detected using ECL Prime (GE Healthcare). Then, the membranes were stripped and reprobed.

### Immunofluorescence

Bouin’s solution-fixed or 4% PFA solution-fixed, paraffin-embedded mouse testes were cut into 8-μm-thick sections and mounted on silane-coated slides. These sections were boiled for 20 minutes in 0.01 M sodium citrate to unmask the epitope. For immunohistochemistry, we used the Envision kit/HRP (DAKO) according to the manufacturer’s protocols. For immunofluorescence, permealization was performed with 0.2% Triton-X in PBS-T for 10 minutes. After blocking with 10% bovine serum albumin for 10 minutes, the sections were probed with primary antibodies at 4°C overnight. The sections were reacted for 1 hour with the secondary antibodies. The primary and secondary antibodies are listed in S4 Table.

### Northern blotting

The freshly removed organs of adult mice were homogenized in RNAzol (Life Technologies). Total RNA samples were extracted according to the manufacturer’s recommendations. Northern blotting was performed according to the manufacturer’s instructions using PerfectHyb (Toyobo). Hybridization was performed by incubating the filter with a 32P-labeled cDNA probe, prepared using the BcaBEST Random Primer Kit (TaKaRa). The signals were detected by STORM 820 (GE Healthcare).

### Luciferase assay

To analyze NFkB activation, 293T cells were co-transfected with 60 μg of pNFkB-Luc and pcDNA-*Nkap* or pcDNA-*Nkapl*. NIH3T3 cells were also co-transfected with pEluc-*CSL* as a reporter plasmid, and pcDNA-NICD1-4 and pcDNA-*Nkap* or pcDNA-*Nkapl* for transcriptional reporter assay of Notch signaling. Fifteen micrograms of pRL-TK was also co-transfected to examine the efficacy of transfection. After 24 hours of incubation, cells lysates with passive lysis buffer were reacted with Dual-Glo Luciferase (Promega). Transcription was measured by expression of luciferin. Data were internally standardized to the amount of luciferase activity from mock. Independent experiments were performed four times.

### Cell culture

GS cells were derived from testicular cells of ICR mice 5 to 7 dpp. Isolation methods and culture conditions were modified referring to descriptions in previous articles [27,28]. Briefly, GS cells were cultured and maintained on mitomycin C-treated mouse embryonic fibroblasts in supplemented Iscov’s modified Dulbecco’s medium with 1% FBS, 50 ul/ml Knockout serum replacement (Gibco) and growth factors including 4 ng/mL recombinant murine endothelial growth factor (Peprotech), 10 ng/mL recombinant human basic fibroblast growth factor (Wako), and 10 ng/mL recombinant murine glial cell-line-derived neurotrophic factor (Peprotech).

### Lentivirus infection

Full-length murine *Nkapl* with FLAG tag (DYKDDDDK) was inserted into CSII-EF-IRES2-puro. It was co-transfected with packaging vector to 293FT cells to produce Lentivirus particles. Lentiviral particles were collected at 48 hours and 72 hours after the transfection. Concentrated lentiviral particles were added to cultured GS cells as previously described. The multiplicity of infection was adjusted from 3 to 5. To isolate GS-Nkapl, infected GS cells were cultured with 0.2 ng/mL puromycin.

### Generation of Nkapl knockout mice

Embryonic stem cell clone with Nkapl deletion was provided from the KOMP Repository (Project ID VG12856). These ES cells (C57/6N) were injected into blastocysts, and these blastocysts were transferred into the uteri of 2.5-dpc pseudopregnant ICR female mice. Mice with mutated alleles were isolated by PCR genotyping using specific primer sets [S5 Table].

### Breeding of the mice

All animal experiments conformed to the Guide for the Care and Use of Laboratory Animals and were approved by the Institutional Committee of Laboratory Animal Experimentation (Nagasaki International University, Nagasaki, Japan) and the Ethics Review Committee for Animal Experimental of Osaka University School of Medicine. Mice were purchased from CLEA Japan, Inc.(Tokyo, Japan) and maintained at 22°C under a 12 hours light-dark cycle (light on from 8:00 to 20:00), provided with food and water ad libitum in the animal experimental facility. In general, mice were sacrificed by overdose intraperitoneal injection of pentobarbital (200mg/kg). Total number of mice for this study was 137. Genomic DNA was extracted from the tails of the mice using standard procedures.

### Statistical analysis

Differences between the experimental and control conditions were compared using the Student *t*-test. A P value of <0.05 was considered to indicate statistical significance.

## Results

### Expression analysis of Nkapl

**Fig 1:**
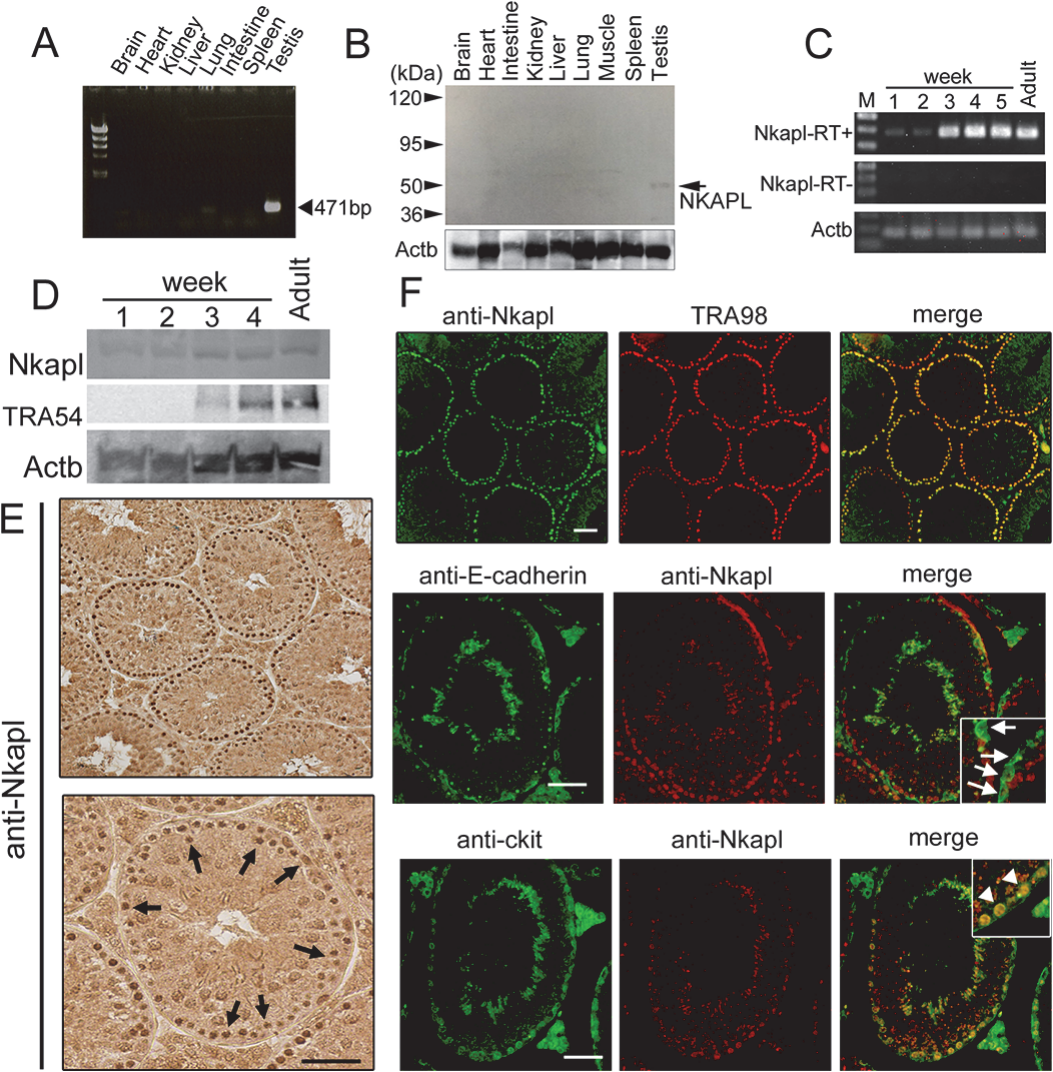
NKAPL expression and localization analyses. (A) RT-PCR analysis of systemic tissue. The targeted amplicon was 471 bp. (B) Immunoblotting analysis of systemic tissue protein. (C) RT-PCR analysis of testis by age. RT+ or RT- represents the presence or absence of reverse transcription, respectively. M: DNA size marker. (D) Immunoblotting of testis by age. (E) Immunohistochemistry of adult testis with Nkapl antibody. Black scale bar = 50 μm. (F) Immunofluorescence of adult testis with Nkapl, TRA98, E-cadherin, and c-kit antibodies. White arrows indicate E-cadherin-positive undifferentiated spermatogonia, and white arrowheads represent c-kit-positive differentiating spermatogonia. White scale bar = 50 μm.

We examined the transcription of *Nkap* and *Nkapl* by reverse transcription polymerase chain reaction (RT-PCR). *Nkap* was transcribed ubiquitously, and *Nkapl* was restricted to testis [Fig 1 and S1 Fig]. To examine the expression and localization of NKAPL protein by immunofluorescence and immunoblotting, we raised murine Nkapl-specific antibody. This antibody showed the specific reaction for the recombinant murine NKAPL protein without any other recombinant proteins [S2 Fig]. Immunoblotting for murine systemic tissues showed a specific reaction for testis at 52 kDa, which was a higher molecular weight than predicted from the putative amino acids of NKAPL. The *Nkapl* gene (accession no.: NM_025719.3) has 1188 base pairs as an open reading frame and its putative molecular weight is 44.86 kDa [Fig 1B].

We examined the change of *Nkapl* expression with age by RT-PCR and immunoblotting. Our findings showed that the expression level was low until the age of 2 weeks, but from 3 weeks on, it was significantly up-regulated, consistent with TRA54 expression [Fig 1C and 1D], of which the antibody is specific from pachytene spermatocytes to spermatids of stages 1–12 [29]. The results of qRT-PCR revealed that the up-regulation after 3 weeks was approximately 50-fold compared with the level at 1 and 2 weeks and maintained high transcription up to the adult (data not shown).

To examine the localization of NKAPL protein in murine testis, immunohistochemistry and immunofluorescence were performed. The results showed that NKAPL was expressed abundantly in spermatogonia and early spermatocytes [Fig 1E]. Further analyses of the localization of NKAPL, consisting of double staining with TRA98, E-cadherin, or c-kit, were performed. TRA98 monoclonal antibody reacts with germ cells, especially highly with spermatogonia [30]. E-cadherin is a family of transmembrane proteins and a marker of undifferentiated spermatogonia including A_single_, A_paired_, and A_aligned_. c-kit is a marker of differentiating spermatogonia, localized from spermatogonia (A_1_ to A_4_, Intermediate, and B spermatogonia) as a transmembrane kinase. NKAPL was recognized in the nuclei of spermatogonia but not in undifferentiated spermatogonia. Robust expression was observed in differentiating spermatogonia, consistent with that of c-kit [Fig 1F]. Taken together, these results indicate that NKAPL is a germ cell-specific protein, which is mainly located to the nuclei of differentiating spermatogonia, with robust expression from 3 weeks of age.

### Analysis of nuclear localization signals in mouse NKAPL

**Fig 2:**
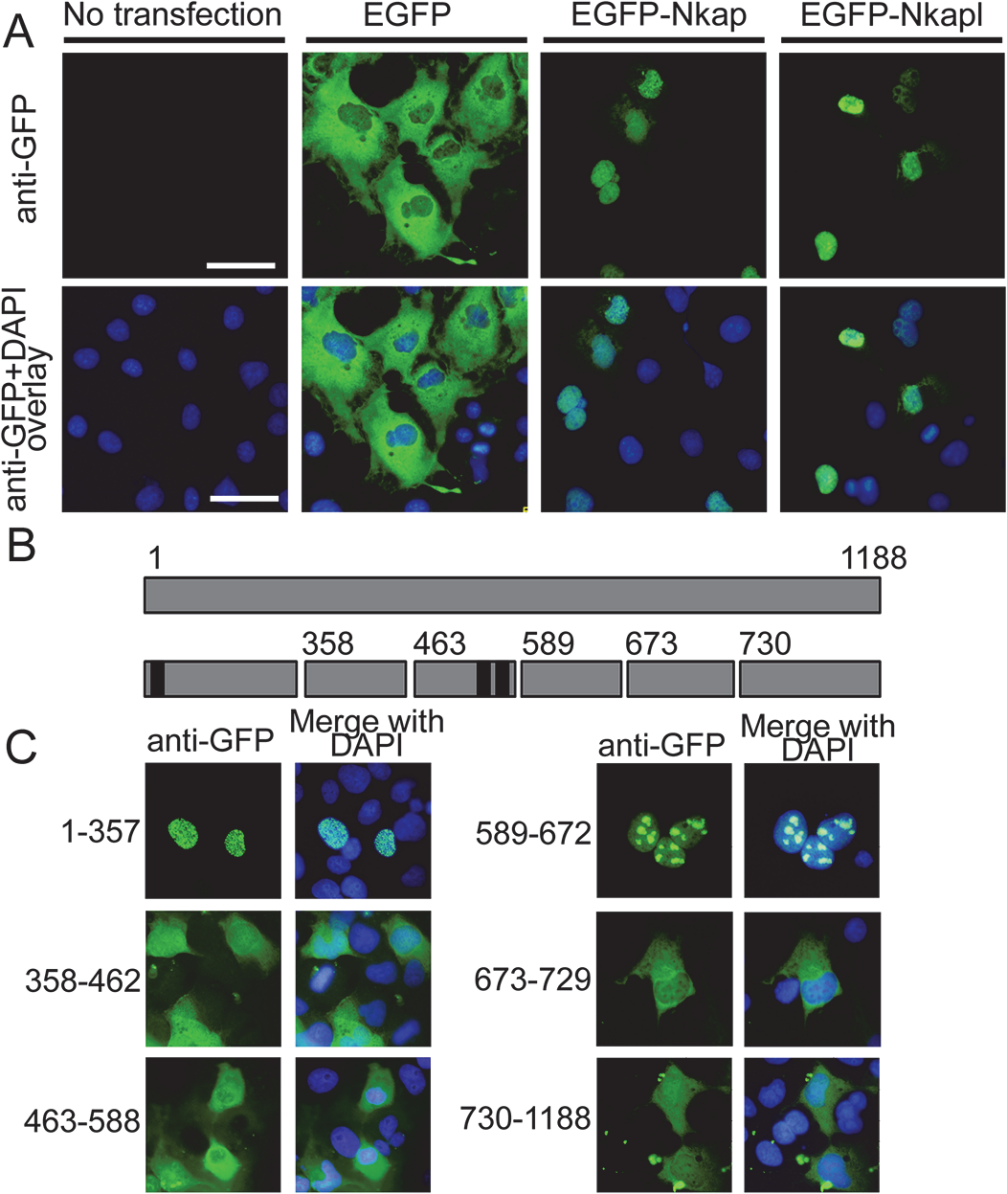
Nuclear localization signal (NLS) analyses of NKAPL *in vitro*. (A) Immunofluorescence with GFP antibody after transfection of EGFP-tagged vectors into 293T cells. Upper row shows images of GFP expression, and lower row shows those merged with DAPI staining to visualize nuclei. White scale bars = 50 μm. (B) The upper bar represents the open reading frame (ORF) of the *Nkapl* gene, and the lower bars illustrate the fragmented sequences of the *Nkapl* gene, which were inserted into EGFP-tagged vectors. Numbers over the bars indicate the fragmented sites on the ORF. Black regions indicate the sequence including the putative NLS signal amino acids predicted by cNLS Mapper prediction program. (C) Immunofluorescence of GFP antibody after the transfection of EGFP-tagged fragmented *Nkapl*. Left columns show the immunofluorescent images with GFP antibody, and the right columns show those merged with DAPI staining.

To analyze the nuclear localization of NKAPL *in vitro*, we transfected 293T cells with *Nkapl*-inserted enhanced green fluorescent protein (EGFP)-expression vector and then performed immunofluorescence. *Nkap*-inserted vector was also used as a positive control. Whereas transfected cells with control vector expressed EGFP in cytoplasm, those with the *Nkap-* and *Nkapl-*inserted vector showed EGFP expression in nuclei [Fig 2A]. These results suggested that translated amino acids of *Nkapl* included nuclear localization signals. Using the cNLS Mapper prediction program for these signals [31], nuclear localization sequences of NKAPL amino acid sequences were predicted as follows: GSQKRRRFSE at the 15th, HSTKKKRKKK at 180th, and KPSKRKHKKYY at 189^th^ [Fig 2B]. To confirm the predicted nuclear localization sequences, we constructed several EGFP expression vectors with insertion of fragmented NKAPL and transfected them into 293T cells. In the case of transfection with expression vector of *Nkapl* fragments including GSQKRRRFSE at 15th, EGFP fusion protein was expressed specifically in the nucleus and was consistent with the expression pattern of transfection with whole *Nkapl*. However, transfection with expression vectors of the other *Nkapl* fragments showed expression of the EGFP in cytoplasm or nucleoli [Fig 2C]. These findings indicated that the amino acids at 15th played a critical role as a nuclear localization signal in NKAPL protein.

### NKAPL protein is a weak activator of NFkB

NKAP was identified as an RIP, which could potentially activate NFkB as well as other RIPs [8]. We hypothesized that NKAPL can also be an activator of NFkB due to its high identity to NKAP. To examine the efficacy of NFkB activation by NKAPL, a luciferase assay was performed by co-transfecting the *Nkapl* expression vector with the reporter gene. As predicted, NKAPL could activate NFkB in a dose-dependent manner to the same extent as NKAP. However, its activation efficacy was weak, and a significant difference in activation efficacy was recognized only when the maximum dose of the *Nkapl* vector was transfected [S3 Fig].

### NKAPL binds to components of, and plays a role as a transcriptional repressor in, Notch signaling

**Fig 3:**
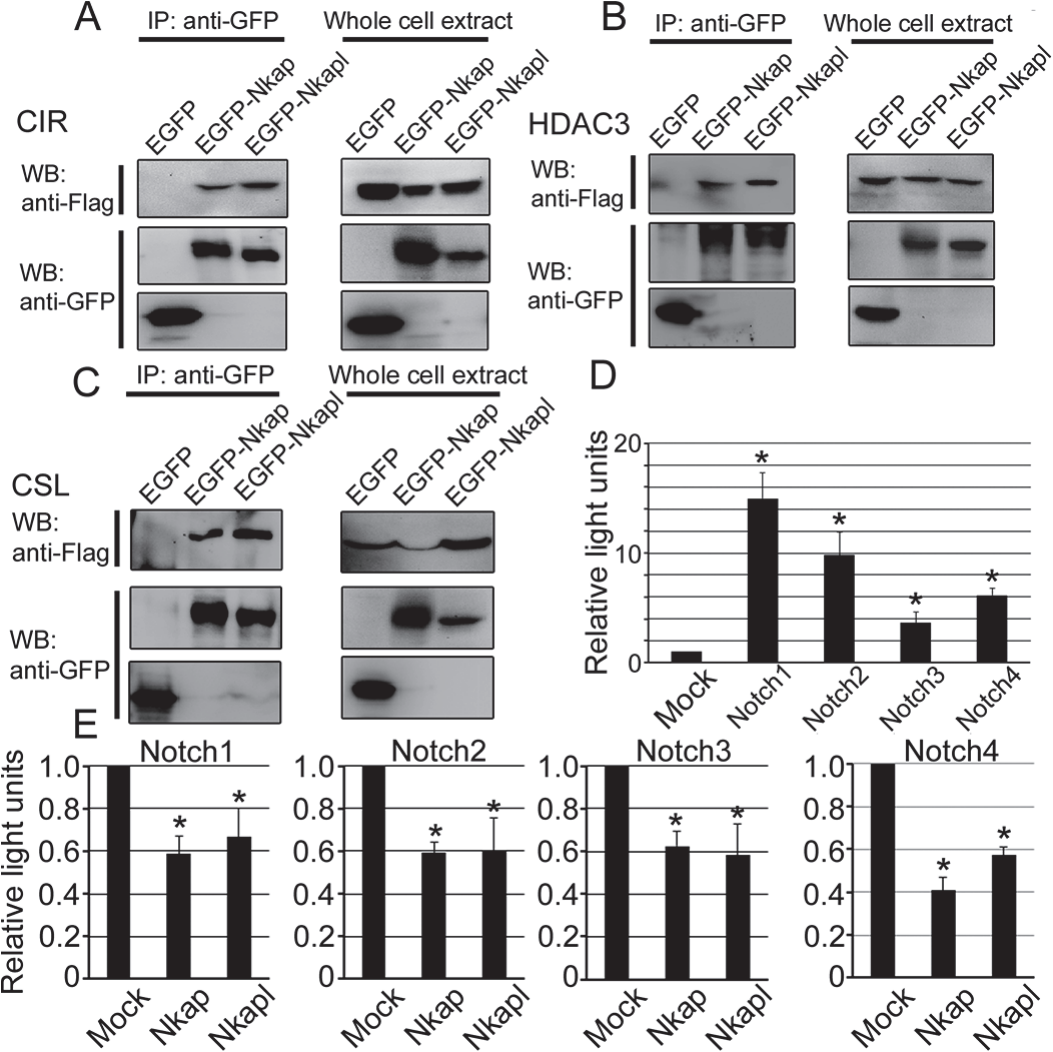
NKAPL interacts with other Notch signaling co-repressors and suppresses downstream transcription of all Notch family receptors. (A-C) interactions between Nkapl and CIR (Corepressor interacting with RBPJ), HDAC3 or CSL (Cbf1/Rbp-jk) by immunoprecipitation (IP) and immunoblotting (WB) with whole-cell extract to confirm expression of co-transfected genes. Nkap was used as positive control. (A) Interaction of NKAP and NKAPL with CIR (Corepressor interacting with RBPJ). (B) Interaction of NKAP and NKAPL with HDAC3. (C) Interaction of NKAP and NKAPL with CSL (Cbf1/Rbp-jk). (D)The Luciferase assay of transcription levels with pEluc-CSL and pcDNA-Notch 1 to 4 co-transfected NIH3T3 cells. Mock represents co-transfection with no insertion of pcDNA vector. (E) The luciferase assay after co-transfection with pEluc-CSL and pcDNA-Notch1 to 4 and pCAGGS-Nkap or Nkapl vectors. Mock represents co-transfection with no insertion of pCAGGS vectors. Error bars show standard deviation (SD) from the means.

In mammals, there are four kinds of Notch transmembrane receptors (Notch1-4) to Notch ligands [10]. On interacting ligands and receptors, Notch receptors cause proteolytic cleavage and subsequently produce NICD. NICD interacts with CSL and initiates the transcription of Notch target genes. Various proteins act on the binding of NICD with CSL, such as CIR and HDAC. NKAP interacts with CIR and HDAC3 [9], and therefore, Nkapl was also considered to associate with these proteins in Notch signaling. To confirm their association, GFP-tagged Nkapl was co-transfected with FLAG-tagged CIR, HDAC3, and CSL, and immunoprecipitated by GFP antibody. All FLAG-tagged CIR, HDAC3, and CSL were detected by anti-FLAG antibody after the immunoprecipitation of EGFP-tagged Nkapl [Fig 3A–3C].

Furthermore, because human NKAP was proven to be a transcriptional repressor in Notch signaling [9], NKAPL can be also considered to have the same function. To examine the function of NKAPL, the *Nkapl* expression vector was cotransfected in NIH3T3 cells with the NICD 1–4 expression vector and the reporter vector with CSL binding domain, and luciferase assays were performed. First, we confirmed the NICD 1–4 activities without NKAP and NKAPL in this assay [Fig 3D]. NICD1 showed the highest activation and NICD3 showed the lowest of the NICD 1–4 vectors, but all NICDs showed significant activity compared to the negative control. Subsequently, we measured the effects on transcription by NKAP and NKAPL overexpression. Both NKAP and NKAPL repressed approximately 40–50% of the transcriptional activities of NICD 1–4 [Fig 3E]. These results suggested that *Nkapl* is a functional transcriptional repressor to the same extent as *Nkap*, interacting with CSL and co-repressor complexes.

### Overexpression of NKAPL activates the Notch signaling pathway and suppresses germ cell differentiation in vitro

**Fig 4:**
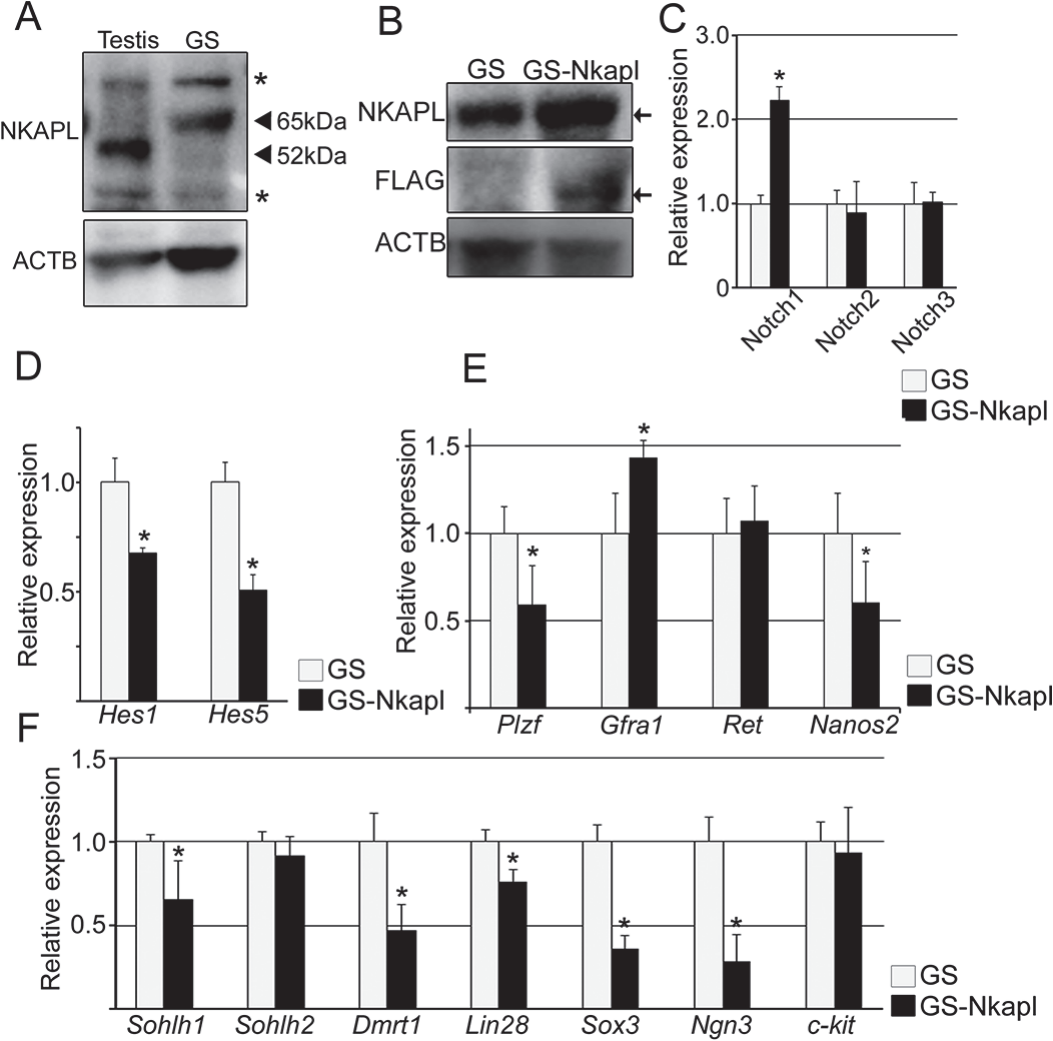
NKAPL suppresses the transcription of downstream target genes through Notch signaling and plays critical roles in spermatogonial stem cell (SSC) maintenance and differentiation. (A) Immunoblotting for proteins extracted from testis or GS cells. Black arrowheads represent the specific reaction for Nkapl antibody. Asterisks represent non-specific reactions. (B) Immunoblotting of GS and GS-Nkapl cells to confirm the expression of the lentiviral-induced *Nkapl* gene. The reaction with FLAG antibody demonstrates its expression. Black arrows represent the specific reactions by each of the antibodies. (C) The transcriptional changes of *Notch1* to *3* between GS and GS-Nkapl cells by qRT-PCR. (D) The transcriptional changes of *Hes1* and *Hes5*. (E) The transcriptional changes of SSC markers. (F) The transcriptional changes of differentiation-associated factors. Error bars indicate standard deviation from the means. *P<0.05.

To examine the function of NKAPL in germ cells, GS cells were applied as an *in vitro* model [27,28]. GS cells were derived from murine pup or adult testes. Their derivation provides us a unique method for studying functional analyses of germ cells (especially SSCs) and germ-cell-specific genes. RT-PCR and immunoblotting showed that NKAPL was expressed in both GS cells and testis [Fig 4A and S4A Fig].

Subsequently, GS cells constitutively overexpressing NKAPL (GS-Nkapl) were isolated by lentiviral induction of *Nkapl* to examine the effect of NKAPL for germ cells [Fig 4B and S4B Fig]. FLAG-tagged NKAPL protein induced by lentiviral infection was observed at 52 kDa, lighter than the endogenous Nkapl protein. GS-Nkapl cells showed a morphological appearance and proliferation in culture comparable with those of the GS cells [S4C Fig].

Although GS cells did not express Notch1 to 3 but Notch4, only *Notch1* showed a significant transcriptional increase by overexpressing Nkapl [S4B Fig and Fig 4C]. *Hes* family genes, which encode basic helix-loop-helix transcriptional repressor, have been considered key genes activated downstream of Notch signaling. The *Hes* family is supposed to execute important aspects of Notch signaling [10]. Especially, Hes1 and Hes5 but other orthologs as well are dependent of the Notch signaling pathway. In GS-Nkapl, transcriptions of *Hes1* and *Hes5* were significantly decreased [Fig 4D]. These findings suggested that overexpressed NKAPL impaired the transcription of the downstream genes through Notch signaling, and *Notch1* was elevated in the form of negative feedback in GS cells.

### Overexpressed NKAPL causes changes in SCC markers and reduces differentiation markers in GS cells

Generally, stem cells have the abilities of both self-renewal and differentiation and maintain a balance between the two. The Notch signaling pathway is a master regulator of cell fate decision in neural and hematopoietic stem cells. Therefore, we hypothesized that alternation of the downstream genes in the Notch signaling pathway would cause changes in the SSC state through the overexpression of NKAPL. To examine this hypothesis, SSC maintenance factors including *Plzf*, *Gfra1*, *Ret*, and *Nanos2* were compared between GS and GS-Nkapl. *Gfra1*, *Ret*, and *Nanos2* are important transcription factors for SSC maintenance induced by GDNF, which is secreted by Sertoli cells [2,32–35]. *Plzf* is a GDNF-independent transcription factor for SSC maintenance and is widely used as a marker of undifferentiated spermatogonia [36]. Analyses by qRT-PCR showed significant elevation of *Gfra1* and decrease of *Plzf* and *Nanos2*, although *Ret* was not changed [Fig 4E]. Subsequently, changes in SSC differentiation transcription factors were examined in GS-Nkapl. A series of these factors are expressed in undifferentiated or differentiating spermatogonia, and they influence the balance between SSC self-renewal and differentiation [37]. All of the differentiation markers except for *Sohlh2* and *c-kit* were significantly decreased by overexpression of NKAPL [Fig 4F]. These findings suggested that NKAPL changed the state of stem cells by suppressing the downstream genes of Notch signaling, and Notch signaling had effects on the balance between differentiation and self-renewal in SSCs.

### Generation of Nkapl-deleted mice and analyses of their phenotypes

**Fig 5:**
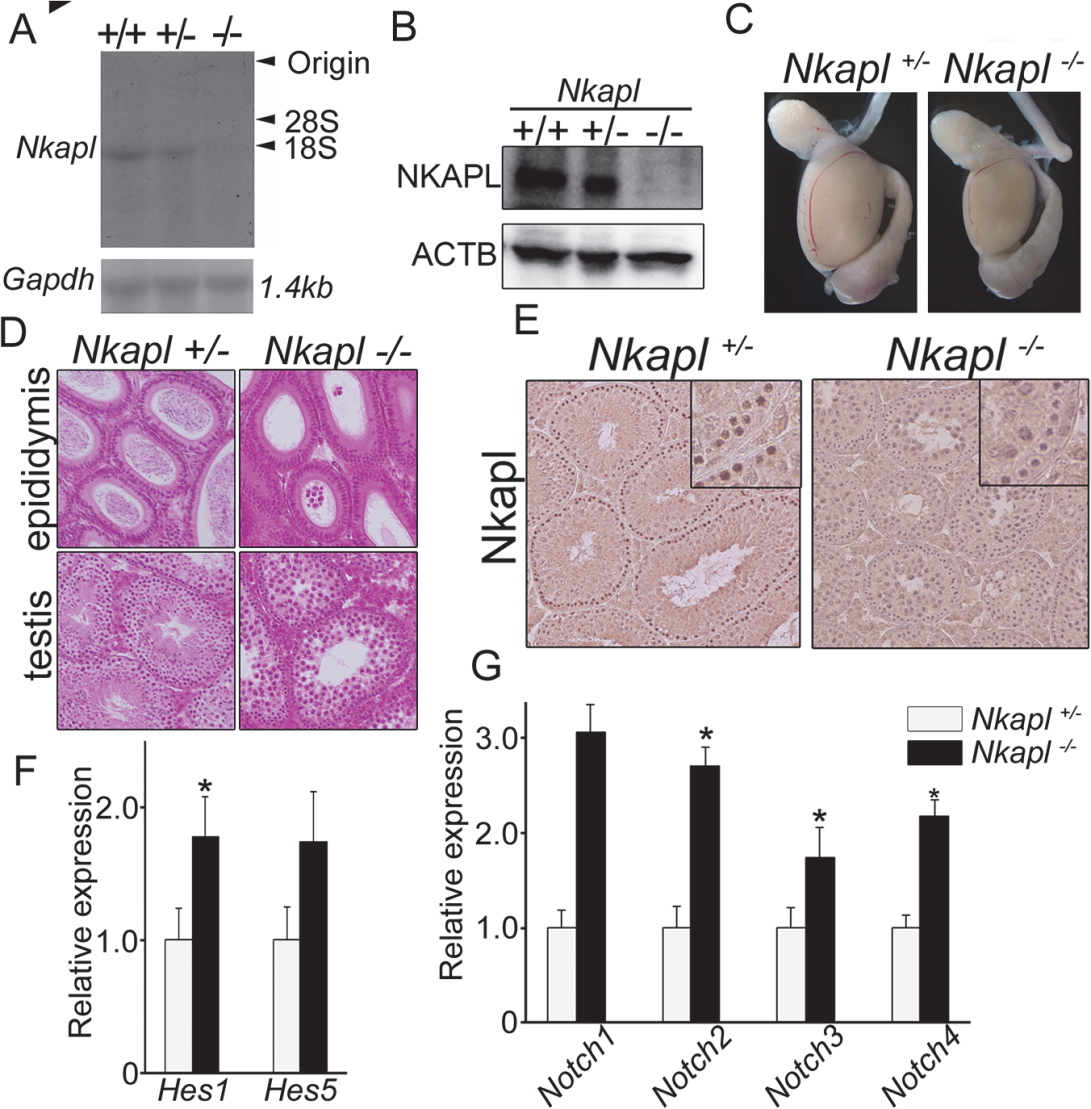
NKAPL deletion caused complete maturation arrest at meiosis through elevation of the Notch signaling pathway. (A) *Nkapl* mRNA transcription in testis of genetically modified mice by Northern blotting. (B) NKAPL expression in testis of genetically modified mice by immunoblotting. (C) Macroscopic appearances of testes in *Nkapl+/-* and *Nkapl-/-* mice. (D) Histology of testis and epididymis stained with hematoxylin and eosin. (E) Immunohistochemistry of Nkapl antibody for testes of *Nkapl+/-* and *Nkapl-/-* mice. (F) Transcriptional changes of *Hes1* and *Hes5* between testes of *Nkapl+/-* and *Nkapl-/-* mice by qRT-PCR. (G) Transcriptional changes of *Notch1 to 4*. Error bars indicate standard deviation from the means. *P<0.05.

We generated *Nkapl*-deleted mice by using the embryonic stem (ES) cells from the KOMP (Knockout Mouse Project, https://www.komp.org) Repository and analyzed their phenotypes to examine the role of *Nkapl* in spermatogenesis[38,39]. The *Nkapl* gene was disrupted, including a part of the open reading frame, and *LacZ* and *neomycin-resistant cassettes (Neo* ^*r*^ *)* were inserted as shown in S5A Fig PCR for genotyping showed the deletion of *Nkapl* and insertion of the *Neo* ^*r*^ *cassette* [S5B Fig]. Transcription of mRNA and the expression of protein were absent in homozygous mice with deleted *Nkapl* [Fig 5A and 5B]. The distribution of the genotypes showed consistency with the expected Mendelian rule of 1:2:1 by confirming the genotypes of offspring generated from crossing heterozygotes (data not shown). *Nkapl* was a germ cell-specific gene, and its homozygous deletion caused no remarkable phenotypic change in the body weight and organs, except in the testis, of the mice [S1 Table]. Male mice with homozygous deletion showed significantly reduced testicular volume [Fig 5C] and infertility [S2 Table], whereas in female mice and their progeny no remarkable phenotypic change occurred. The histology of the seminiferous tubules with homozygous deletion was that of complete meiotic arrest with no haploid cells [Fig 5D and 5E]. No spermatozoa were recognized in the epididymis, and all germ cells were arrested at the pachytene spermatocyte stage.

*Hes1* and *Hes5*, which are some of the downstream genes involved in Notch signaling, were analyzed to examine the effect of *Nkapl* deletion. Although both genes were elevated, only the increase in *Hes1* showed a significant difference [Fig 5F]. Intriguingly, the entire Notch family showed significantly elevated transcription [Fig 5G]. Taken together, *Nkapl* has an indispensable role in spermatogenesis through the Notch signaling pathway *in vivo*, suggesting that aberrant elevation of Notch signaling can disrupt spermatogenesis.

### Germ cell maturation arrest and induction of apoptosis at the level of pachytene spermatocytes in Nkapl-deleted mice

**Fig 6:**
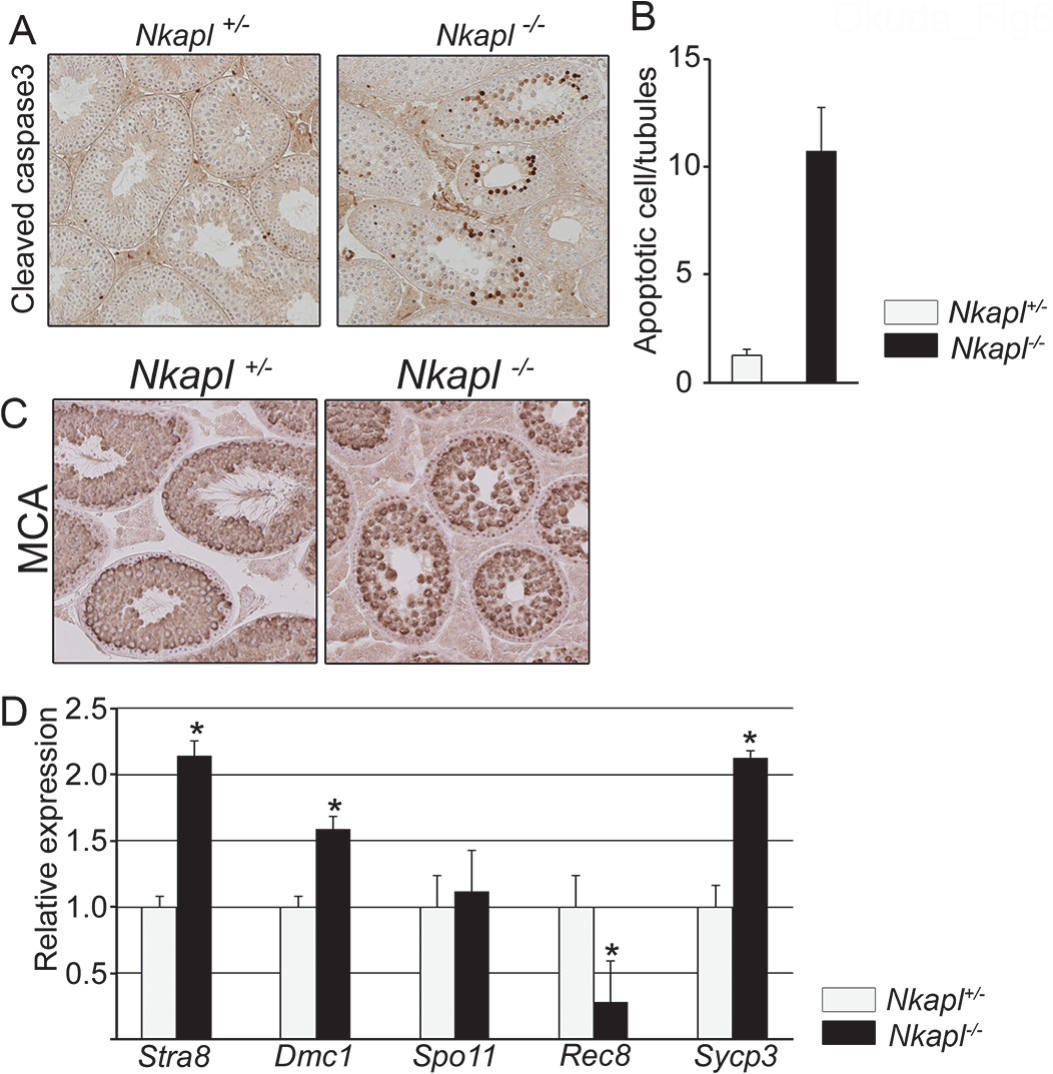
*Nkapl* deletion caused significant apoptosis at the level of pachytene spermatocytes due to aberrant changes of meiosis-related genes. (A) Immunohistochemistry of cleaved caspase-3 antibody for testes of *Nkapl+/-* and *Nkapl-/-* mice to detect apoptotic cells. (B) The average counts of apoptotic cells per seminiferous tubule. One hundred seminiferous tubules were randomly selected, and cleaved caspase-3-positive germ cells were counted. Error bars are standard deviation (SD) from the means. (C) Immunohistochemistry of MCA in testes of *Nkapl+/-* and *Nkapl-/-* mice. (D) Transcriptional changes of meiosis-related genes between testes of Nkapl^+/-^ and Nkapl^-/-^ mice by qRT-PCR. Error bars indicate SD from the means. *P<0.05.

A complete and specific arrest at the level of pachytene spermatocytes in epithelial stage IV was first described in a number of genetically modified mice [40]. At this level, aberrant spermatocytes would induce apoptosis and would be removed as a quality checkpoint [2]. To confirm whether the complete meiotic arrest was caused by apoptosis, qRT-PCR of apoptosis-associated genes was performed. Each of the relative expression levels of adult mice with *Nkapl* deletion was compared with those of prepubertal mice around 10 days postpartum (dpp) because the expression of *Nkapl* increased significantly after 3 weeks of age. Significant increases in expression were observed at *p53*, *Bcl2*, *Bax*, *Fas*, and *Fasl* [S6 Fig]. Immunohistochemistry of cleaved caspase-3 antibody showed an approximately 10-times higher rate of apoptosis in homozygous mice with *Nkapl* deletion [Fig 6A and 6B]. Immunohistochemistry using MCA (Meichroacidin) antibody was conducted to confirm at which stage the apoptosis was induced. MCA is expressed predominantly from early spermatocytes to round spermatids [41]. The results revealed that germ cells with *Nkapl* deletion arrested at the level of pachytene spermatocytes [Fig 6C], which were extremely sensitive to the abnormality of germ cell development. Genetically modified mice often demonstrate meiotic arrest. As one of the systems to guarantee the quality of meiotic cells, the pachytene checkpoint prevents meiotic nuclear division of abnormal cells, which are subsequently removed by apoptosis [42]. *Stra8* is expressed in spermatogonia and spermatocytes (preleptotene) and regulates meiotic initiation by retinoic acid. *Stra8*-deleted mice produce no sperm due to germ cell apoptosis at meiotic prophase [43]. Furthermore, *Stra8* is required for germ cells to exhibit the molecular hallmarks of meiotic chromosome cohesion, synapsis, and recombination including *Dmc1*, *Spo11*, *Rec8*, and *Sycp3* [44]. *Spo11* and *Rec8* are involved in DNA double-strand break formation and homologous recombination during meiosis [45–47], whereas *Dmc1* encodes a recombinase functioning in meiotic double-strand break repair and plays a central role in homologous recombination [48,49]. *Sycp3* is a structural component of the axial/lateral element of the synaptonemal complex [50]. The aberrant expression of these genes may lead to meiotic failure or meiotic arrest by abnormal meiosis and apoptosis [42,51]. We examined the changes of these factors by qRT-PCR to assess the association between the apoptosis of pachytene spermatocytes and the pachytene checkpoint. Significant increases of *Stra8*, *Dmc1*, *and Sycp3*, and a decrease of *Rec8* were proven, indicating that the aberrant meiotic process caused by *Nkapl* deletion undergoes checkpoint-mediated arrest at the level of pachytene spermatocytes [Fig 6D].

### Nkapl deletion causes transcriptional changes in SSC markers and spermatogonial differentiation in mice

**Fig 7:**
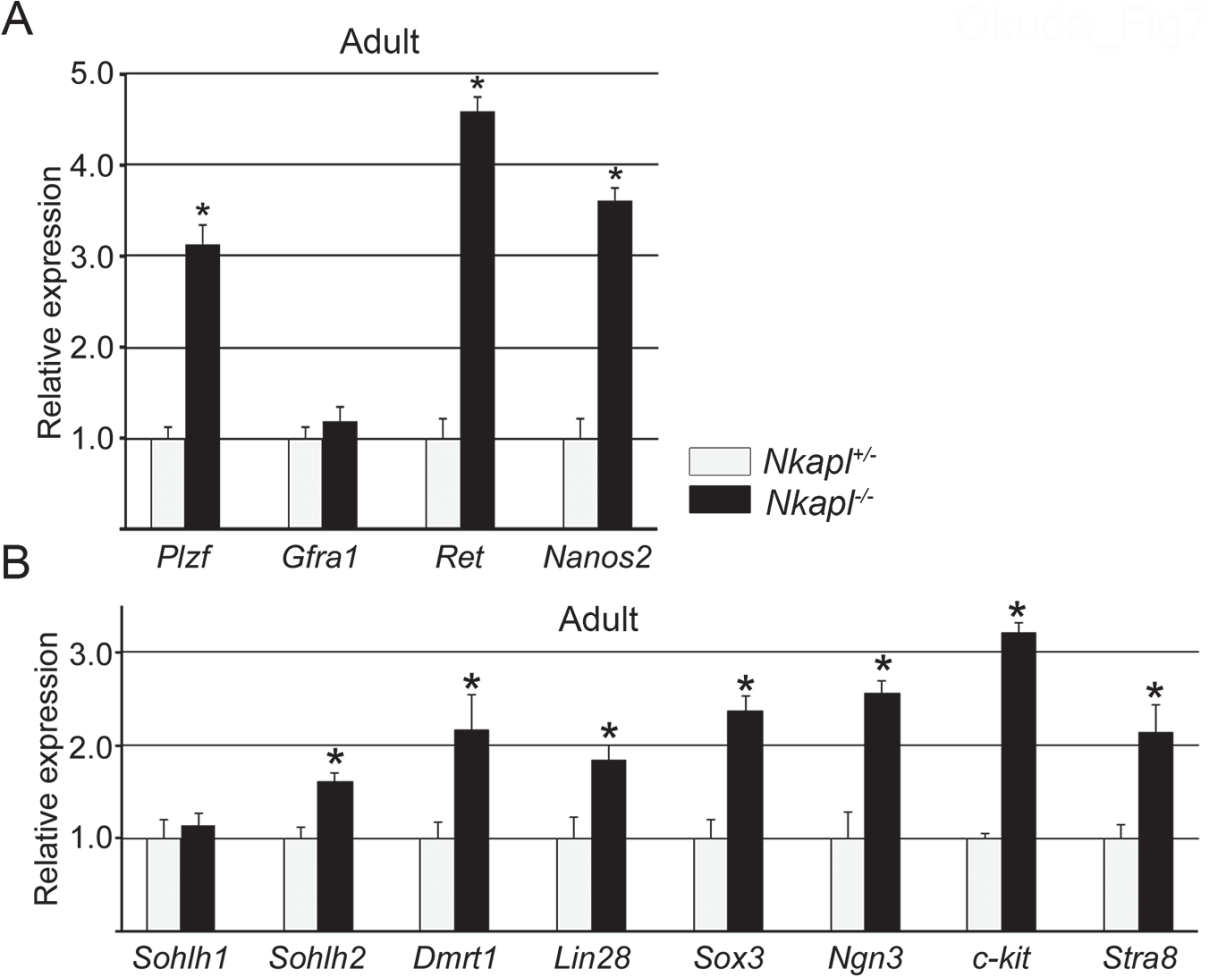
*Nkapl* deletion caused significant changes in spermatogonial stem cell (SSC) maintenance markers and increases in differentiation-related factors. (A) Transcriptional changes of SSC maintenance markers between testes of adult Nkapl^+/-^ and Nkapl^-/-^ mice by qRT-PCR. (B) Transcriptional changes of differentiation-related factors. Error bars indicate standard deviation from the means. *P<0.05.

Finally, we analyzed the effect of *Nkapl* deletion on SSC markers and differentiation *in vivo* because *Nkapl* overexpression in GS cells caused transcriptional changes in SSC markers and differentiation. We examined the transcriptional changes of SSC markers and differentiation markers in both 10-dpp and adult murine testes with heterozygous or homozygous *Nkapl* deletion. There was no significant change in the 10-dpp testes in terms of SSC markers [S7A Fig], but in adult testes, SSC markers except for *Gfra1* were significantly increased [Fig 7A]. Unexpectedly, *Gfra1* changed in GS-Nkapl but not in *Nkapl*-deleted testes, whereas *Ret* was increased in Nkapl-deleted testes but not in GS-Nkapl [Fig 4E]. In differentiation, although not changed in 10-dpp testes [S7B Fig], all factors but *Solhl1* showed significant transcriptional elevation [Fig 7B]. These findings suggest that *Nkapl* had a critical effect on SSC maintenance and germ cell differentiation *in vivo*, comparable to the results of GS-Nkapl *in vitro*.

## Discussion

In this study, we demonstrated that a novel germ cell-specific gene *Nkapl* is expressed in spermatogonia and spermatocytes and affects germ cell differentiation and SSC maintenance through the Notch signaling pathway. *Nkapl* is indispensable for normal spermatogenesis because its disruption caused maturation arrest at the level of pachytene spermatocytes by apoptosis.

NKAPL had similar function to NKAP *in vitro* due to its high identity to Nkap, but its expression in testis was different from that of NKAP, which was localized to post-meiotic germ cells (data not shown). In fact, some retrotransposed genes such as *Phosphoglycerate Kinase2* (*PGK2*) [52], *Glyceraldehyde 3-phosphate dehydrogenase-S* (*GAPDH-S*) [53], and *Phosphoglycerate Mutase4* (*PGAM4*) [4] are testis-specific and essential, compensating for the function of the parent gene by converting the expression pattern before and after meiosis. Notch signaling components (NOTCH1-3) and their downstream effectors (HES1 and HES5) have a unique expression pattern along spermatogenic cycles in neonatal and adult mice [24]. Spermatogonia expressed NOTCH1 and NOTCH3, and NOTCH2 expression started from preleptotene spermatocytes in adult testis. We demonstrated that overexpression of Nkapl significantly increased NOTCH1 but not NOTCH2 and 3 in GS cells. Furthermore, it had the remarkable effect of reducing differentiation factors and changing SSC maintenance markers. These results imply that NOTCH1 mainly plays a critical role in the balance of self-renewal and differentiation in SSC. Furthermore, *Nkapl*-deleted mice showed elevated expression of all Notch family members. Nkapl has suppressive ability for all Notch family members, and its disruption can cause abnormal effects for all germ cells associated with Notch signaling for their differentiation. We can assume that NKAPL has different effects on each differentiation stage by suppressing the different type of Notch because the localization and expression patterns of NOTCH1-4 are different by each stage.

The Notch signaling pathway is essential for the regulation of cell fate during development and throughout postnatal life in self-renewing tissues. In T-cell development, NOTCH1 deficiency causes a developmental block at an early stage. However, constitutional high expression of NOTCH1 by induction of the *Notch1* active domain [54] or deletion of *Nkap*, a transcriptional suppressor of the Notch signaling pathway [9], also blocked T-cell development. These findings suggested that Notch signaling should be controlled properly to sustain T-cell development, and both constitutional activation and complete inactivation of Notch signaling hamper its development.

In germ cells, some reports noted that Notch signaling is dispensable for normal spermatogenesis [21,22]. Histology of seminiferous tubules in mice with deletion of *Notch1* and *Pofut1*, a fucosyltransferase that activates all Notch receptors by transferring fucose to the Notch extracellular domain, showed normal spermatogenesis. Meanwhile, mice with *Notch1* gain of function showed significantly decreased spermatogenesis and increased apoptosis of germ cells as they aged [23]. These findings suggested that only abnormal activation of the Notch pathway could lead to the impairment of spermatogenesis. Our data are compatible with this hypothesis. In fact, NKAPL was expressed continuously in postnatal testis, but its transcription was elevated by 50-fold after 3 weeks of age. Furthermore, *Notch1-3* transcriptional levels in postnatal testis showed interesting changes along with age [S8 Fig]. *Notch1* transcription reached a peak at 2 weeks of age and then was reduced significantly as the mice aged. *Notch2* and *3* gradually decreased with age. These results were consistent with the timing of NKAPL elevation in postnatal testis and support the hypothesis in regard to Notch signaling for spermatogenesis.

Although we proved that the modifications of *Nkapl* expression level could change Notch signaling downstream target genes in germ cells, surprisingly, *Hes1* and *Hes5* were changed less than expected both *in vitro* and *in vivo*. In fact, *Nkap*-deficient thymocytes from *Nkap* ^fl/o^ *Lck* ^Cre^ exhibit much higher increases, such as 20-fold in *Hes1*, 10-fold in *Deltex1*, and 8-fold in *CD25* [9]. One possibility is that the Notch signaling pathway has another stronger effector in germ cells other than the *Hes* family because NICD has been previously reported to have a variety of downstream target genes and to affect other signaling pathways such as *Cd25*, *Cdkn1a*, and *Deltex* in somatic cells [55]. Although these factors can also be candidates of target genes in germ cells, it is not well known which factors are expressed and function in germ cells. Further studies to identify which factor is the most relevant downstream target gene of Notch signaling in germ cells will be needed.

*Nkapl*-deficient mice demonstrated elevation of differentiation factors and some characteristic changes of SSC maintenance markers. Our data on overexpression of NKAPL in GS cells inversely supported this result, showing compatible changes of several factors. These findings imply that proper regulation of Notch signaling is important in sustaining the balance of spermatogonial self-renewal and differentiation. However, with which factors or signaling pathways are Notch signaling and NKAPL relevantly associated? SSC maintenance and differentiation are very complex regulated system, and each factor can activate or repress several other factors and signaling pathways [37]. Our results revealed that overexpression of Nkapl suppressed some of the transcriptional factors associated with germ cell differentiation such as *Sohlh1*, *Dmrt1*, *Lin28*, *Sox3*, and *Ngn3*. Of these factors, *Lin28*, *Sox3*, and *Ngn3* are expressed predominantly in undifferentiated spermatogonia, whereas *Sohlh1* and *Dmrt1* are expressed in differentiating spermatogonia. Some previous reports noted that *Sox3*, *Sohlh1*, and *Sohlh2* stimulated *Ngn3*, which could be a good candidate as a central hub of SSC differentiation by being regulated by various other known differentiation factors [56,57]. Another report stated that *DMRT1* acted in activating transcription of *SOLHL1*, promoting differentiation [58]. Although the relations of these factors are not fully elucidated, *Dmrt1* and *Sox3* can be candidate genes affected by the modification of Notch signaling and *Nkapl* in germ cells. Further study of the correct identification of the Notch signaling downstream genes and other target genes or signaling pathways related to SSC maintenance and differentiation is necessary.

Recent studies have revealed that genetic abnormalities might affect the function or expression of indispensable proteins for spermatogenesis and thereby adversely affect male fertility [59,60]. Thus, finding a novel functional gene and analyzing its role in spermatogenesis will contribute to understanding the complicated mechanisms involved in spermatogenesis and elucidating the causes of male infertility. In our study, *Nkapl-*deleted mice showed typical maturation arrest at meiosis. Therefore, the possibility that genetic mutations or polymorphisms in human NKAPL are associated with human male infertility is being examined.

## Supporting Information

S1 Fig RT-PCR analysis using systemic tissues. The targeted amplicon was 744 bp.
(TIF)

S2 Fig Nkapl antibody raised by the murine NKAPL peptide ESPPSALQTSRSPR was specifically reacted to only murine NKAPL protein. 293T cells were transfected with FLAG-tagged insertion vectors, and extracted proteins were detected with the NKAPL or FLAG antibody by immunoblotting. Mock represents the transfection with no inserted vector.
(TIF)

S3 Fig NKAPL is a weak activator for NFkB. 293T cells were co-transfected with pNFkB-Luc and expression vectors. Control represents the sample co-transfected with no insertion expression vectors. Error bars indicate standard deviation from the means. *P<0.05.
(TIF)

S4 Fig NKAPL and Notch family are expressed in germline stem (GS) cells, and GS cells with NKAPL overexpression (GS-Nkapl) were isolated. (A) RT-PCR of *Nkapl* using DNaseI-treated RNA from GS cells. RT+ or RT- represents the presence or absence of reverse transcription, respectively. M: DNA size marker. (B) *Nkapl* transcription levels between GS cells and GS-Nkapl were compared by qRT-PCR. (C) Morphological appearance by phase contrast microscopy. Both GS cell lines proliferated in morula-like clumps. (D) Quantification of Notch family expression on GS cells. The results were equalized by volume of GS mRNA. *P<0.05.
(TIF)

S5 Fig Targeted Nkapl genomic region and genotyping for generating Nkapl-deleted mice. (A) Illustrations of the targeted genomic region and inserted cassette by homologous recombination. Vertical black arrows with numbers represent the BamHI-specific restriction sites. Horizontal black arrows with letters are targeted sites of primers for genotyping PCR. (B) The *ZEN-Ub1 cassette* insertion was confirmed by PCR using primer sets of B-C and F-G. Identification of the *neomycin-resistant gene* insertion and *Nkapl* genome deletion in mice with *Nkapl*-deleted alleles was confirmed by PCR and sequencing using primer sets of A-E and H-D.
(TIF)

S6 Fig Changes in apoptosis-related genes in testes of *Nkapl* deleted mice. Transcriptional changes of apoptosis-related genes between testes of 10-days postpartum (dpp) (A) and adult (B) *Nkapl* ^*+/-*^ and *Nkapl* ^*-/-*^ mice by qRT-PCR. Expressions in *Nkapl* ^*+/-*^ mice were assumed to equal 1. Error bars indicate standard deviation from the means. *P<0.05.
(TIF)

S7 Fig *Nkapl* deletion showed no changes in spermatogonial stem cell (SSC) maintenance markers and differentiation-related factors in 10-days postpartum (dpp) mice. Transcriptional changes of SSC maintenance markers (A) and differentiation-related factors (B). Error bars indicate standard deviation from the means. *P<0.05.
(TIF)

S8 Fig Transcriptional changes of *Notch1-3* along with age by qRT-PCR. The expressions at 1 week were assumed to equal 1. Error bars indicate standard deviation from the means. *P<0.05.
(TIF)

S1 Table Fertility rates among the mutant mice. (DOCX)

S2 Table Body and organ weights of the *Nkapl* knockout mice. All values are means ± SEM. Significant differences (P<0.01) are discussed here.
(DOC)

S3 Table qRT-PCR primers. (XLSX)

S4 Table List of antibodies. (XLSX)

S5 Table Sequences list for genotyping. PCR was performed using Gflex DNA polymeraseTaq (Takara, Shiga, Japan). Cycling conditions were: 94ºC for 2 min, followed by 35 cycles of denaturation at 98ºC for 10 s, annealing and extension at 68ºC for 1 min.
(XLSX)
