# Supplementary figures and images for: A Novel Transcriptional Factor Nkapl Is a Germ Cell-Specific Suppressor of Notch Signaling and Is Indispensable for Spermatogenesis
Source: PLoS One. 2015 Apr 14;10(4):e0124293. doi: 10.1371/journal.pone.0124293 (PMC4397068; doi:10.1371/journal.pone.0124293)

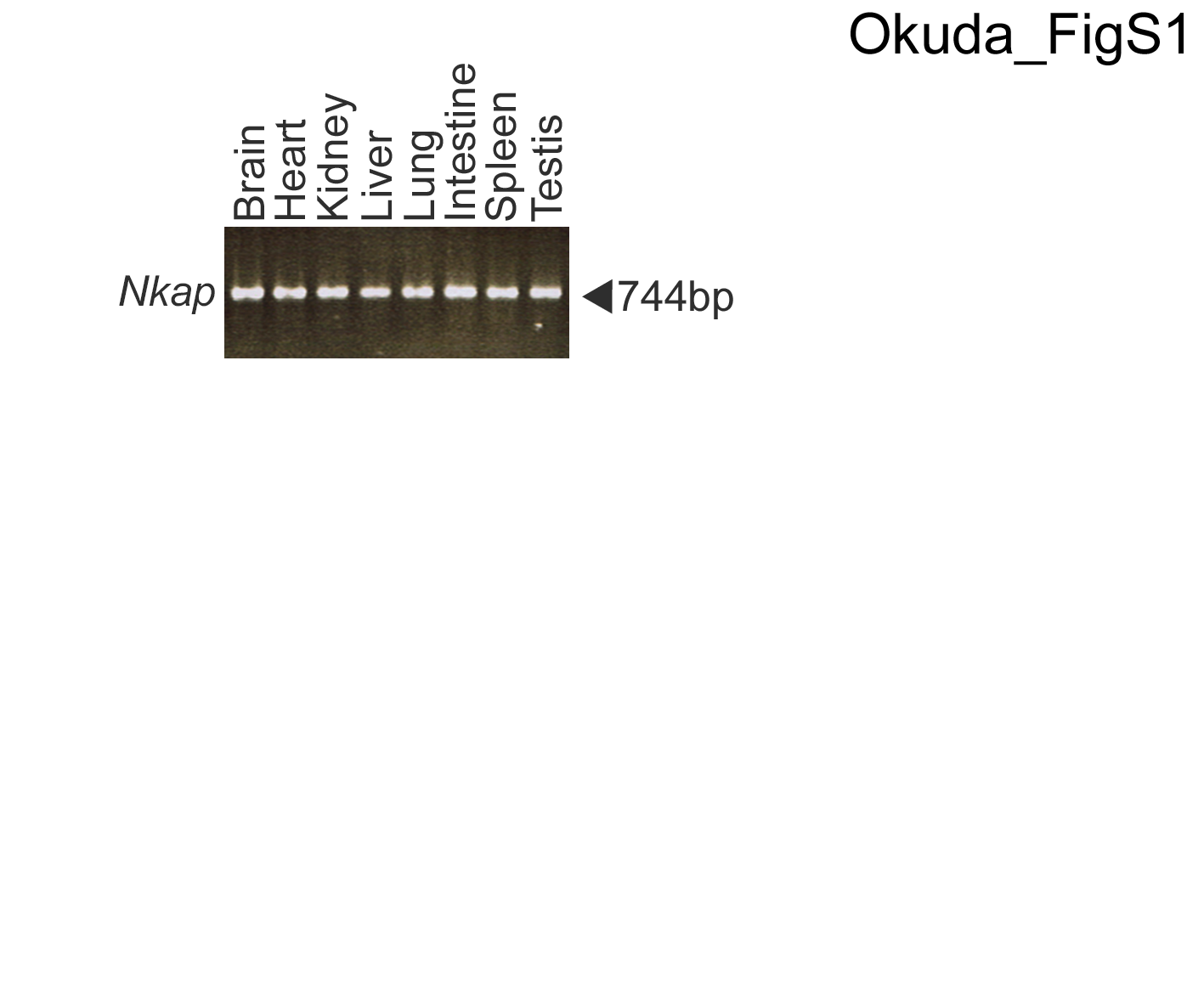

Supplement: S1 Fig — The targeted amplicon was 744 bp. (TIF) [file pone.0124293.s001.tif]

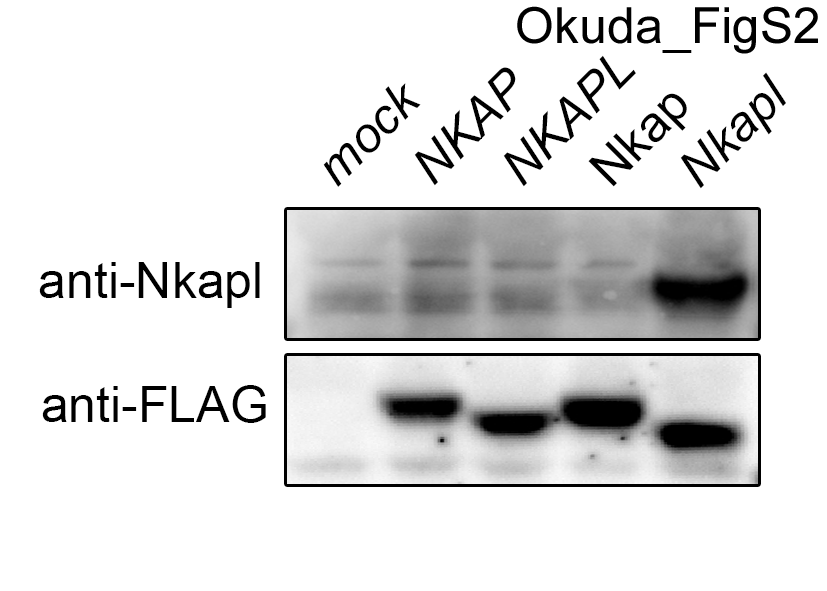

Supplement: S2 Fig — 293T cells were transfected with FLAG-tagged insertion vectors, and extracted proteins were detected with the NKAPL or FLAG antibody by immunoblotting. Mock represents the transfection with no inserted vector. (TIF) [file pone.0124293.s002.tif]

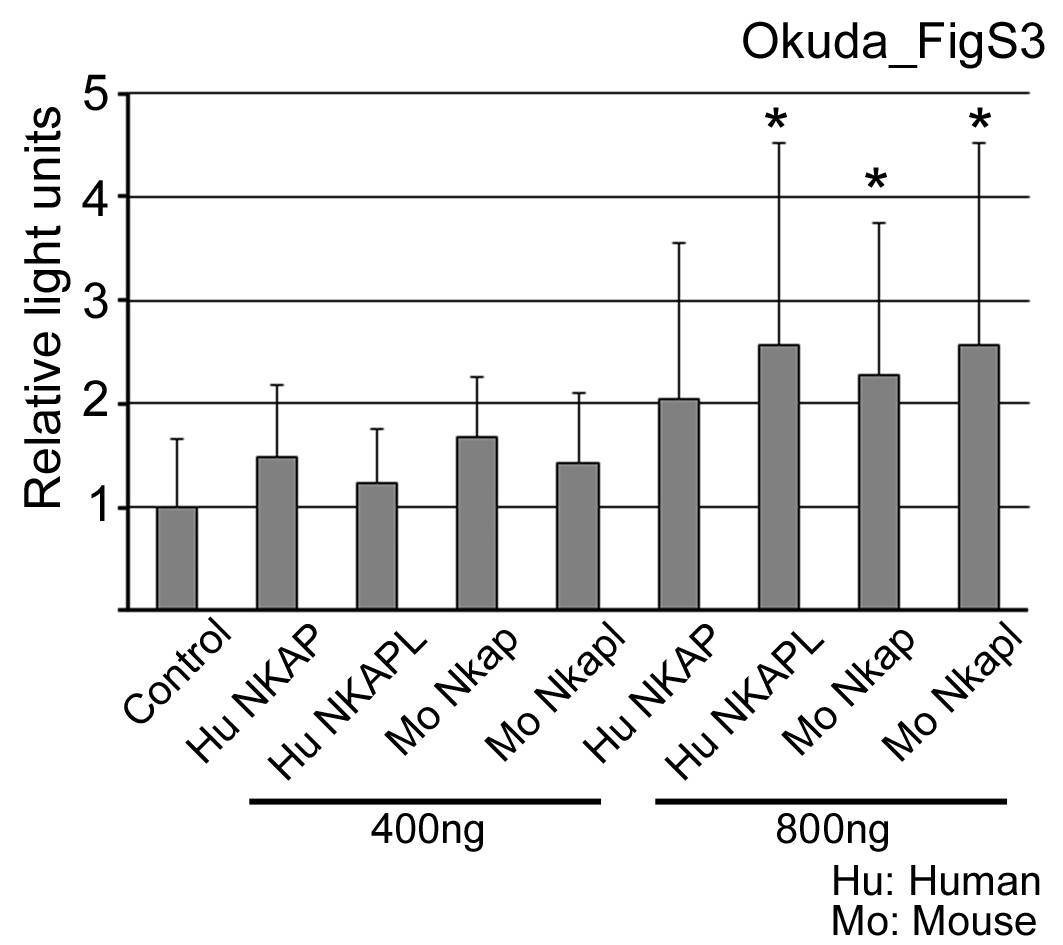

Supplement: S3 Fig — 293T cells were co-transfected with pNFkB-Luc and expression vectors. Control represents the sample co-transfected with no insertion expression vectors. Error bars indicate standard deviation from the means. *P<0.05. (TIF) [file pone.0124293.s003.tif]

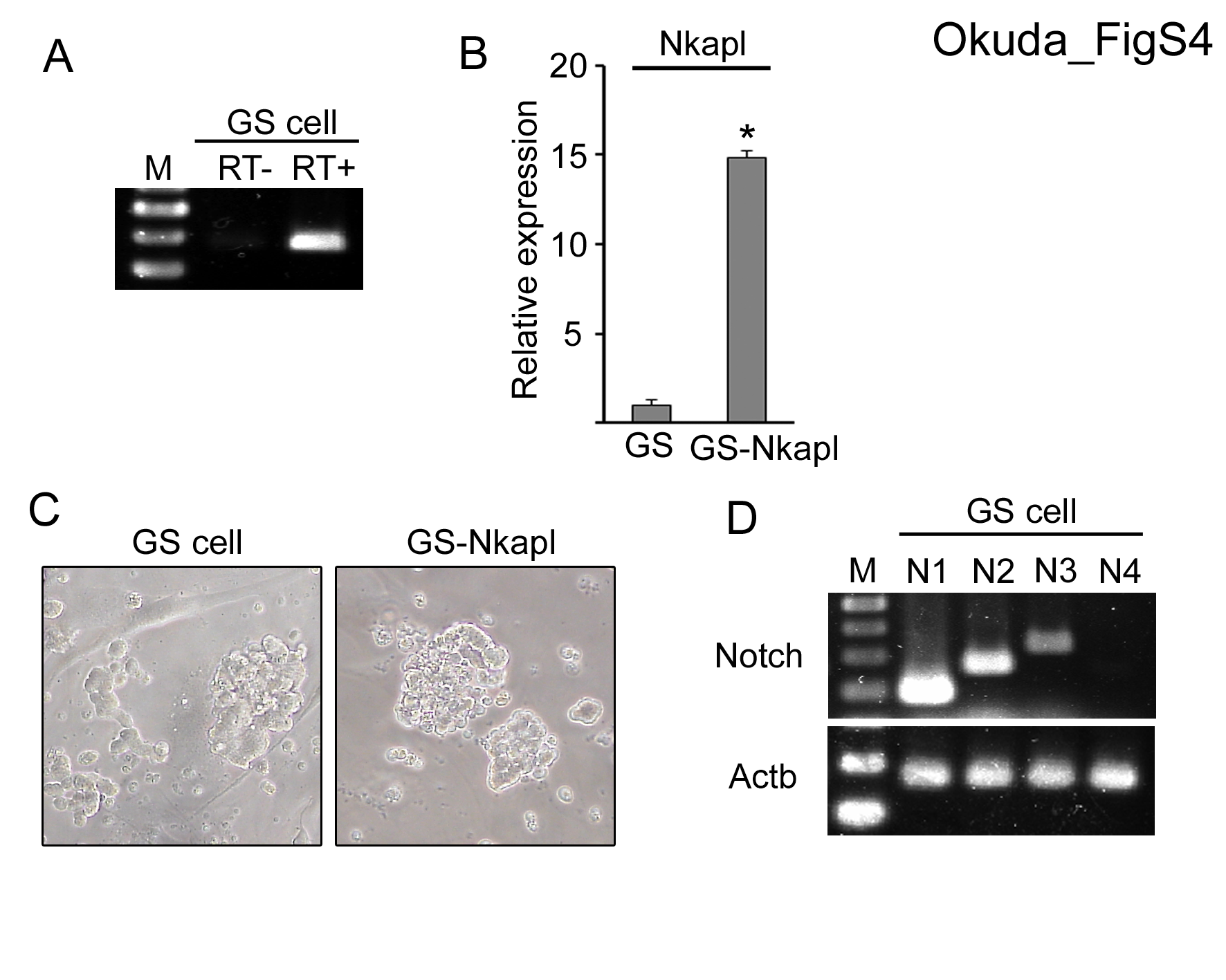

Supplement: S4 Fig — (A) RT-PCR of Nkapl using DNaseI-treated RNA from GS cells. RT+ or RT- represents the presence or absence of reverse transcription, respectively. M: DNA size marker. (B) Nkapl transcription levels between GS cells and GS-Nkapl were compared by qRT-PCR. (C) Morphological appearance by phase contrast microscopy. Both GS cell lines proliferated in morula-like clumps. (D) Quantification of Notch family expression on GS cells. The results were equalized by volume of GS mRNA. *P<0.05. (TIF) [file pone.0124293.s004.tif]

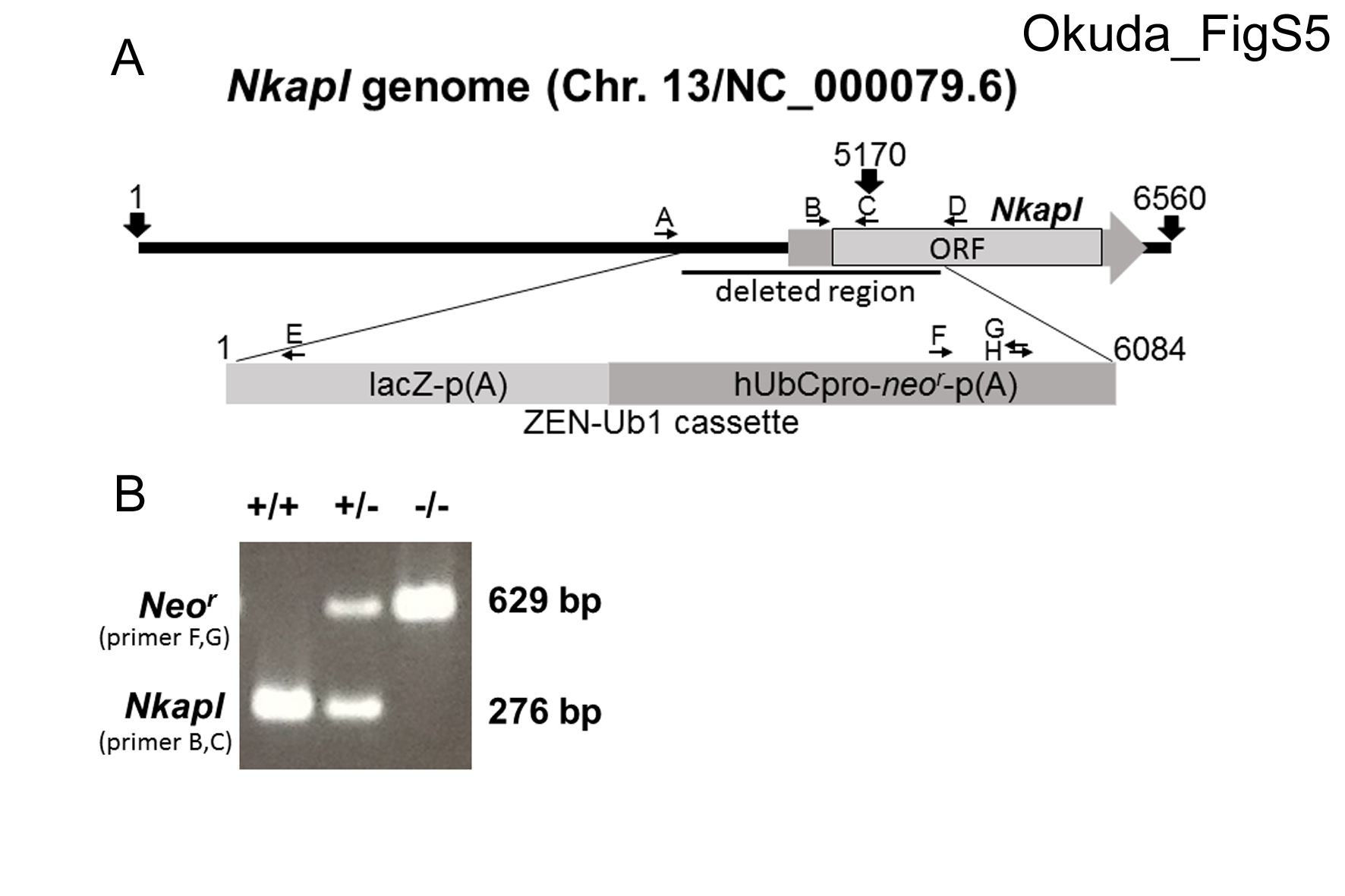

Supplement: S5 Fig — (A) Illustrations of the targeted genomic region and inserted cassette by homologous recombination. Vertical black arrows with numbers represent the BamHI-specific restriction sites. Horizontal black arrows with letters are targeted sites of primers for genotyping PCR. (B) The ZEN-Ub1 cassette insertion was confirmed by PCR using primer sets of B-C and F-G. Identification of the neomycin-resistant gene insertion and Nkapl genome deletion in mice with Nkapl-deleted alleles was confirmed by PCR and sequencing using primer sets of A-E and H-D. (TIF) [file pone.0124293.s005.tif]

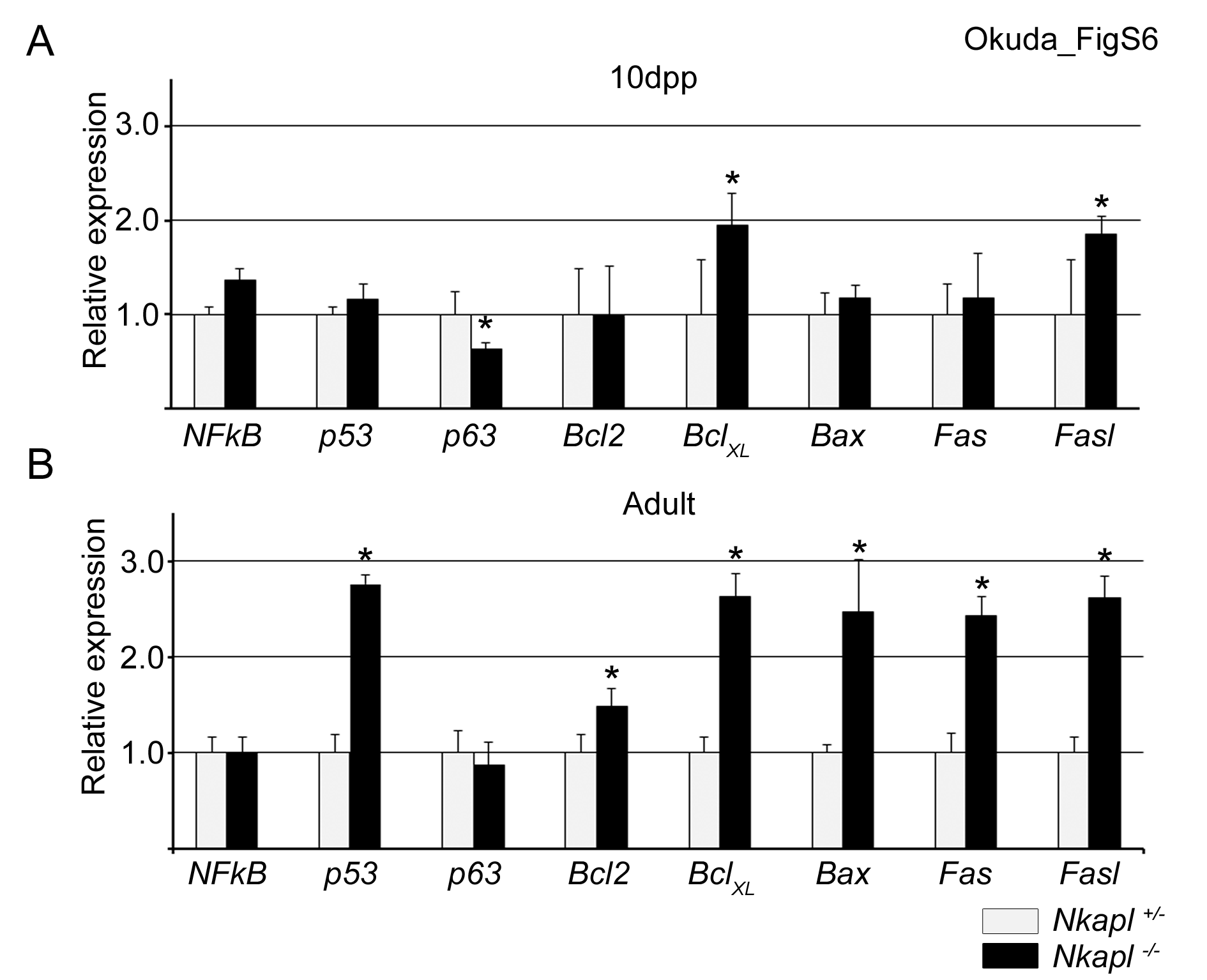

Supplement: S6 Fig — Transcriptional changes of apoptosis-related genes between testes of 10-days postpartum (dpp) (A) and adult (B) Nkapl +/- and Nkapl -/- mice by qRT-PCR. Expressions in Nkapl +/- mice were assumed to equal 1. Error bars indicate standard deviation from the means. *P<0.05. (TIF) [file pone.0124293.s006.tif]

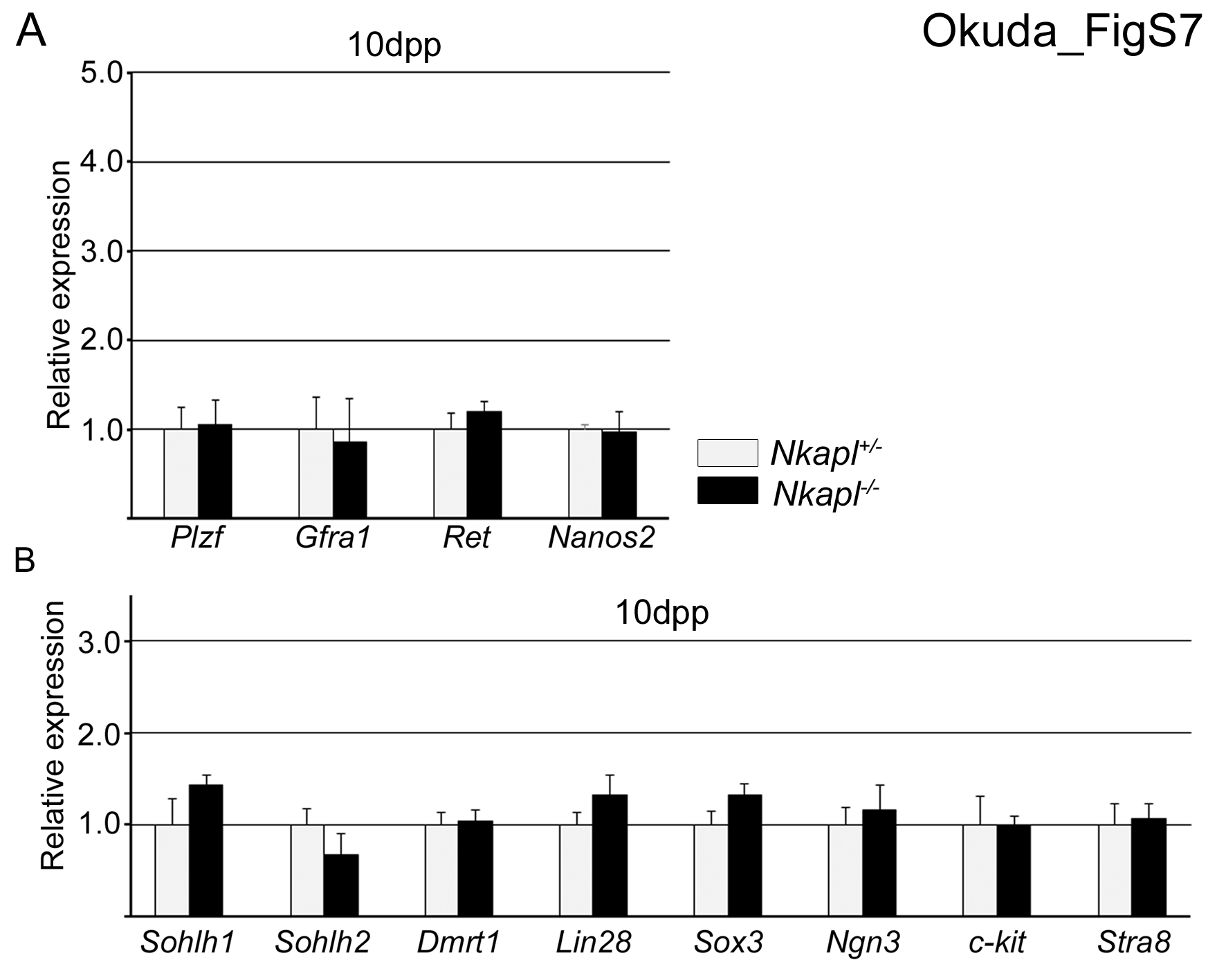

Supplement: S7 Fig — Transcriptional changes of SSC maintenance markers (A) and differentiation-related factors (B). Error bars indicate standard deviation from the means. *P<0.05. (TIF) [file pone.0124293.s007.tif]

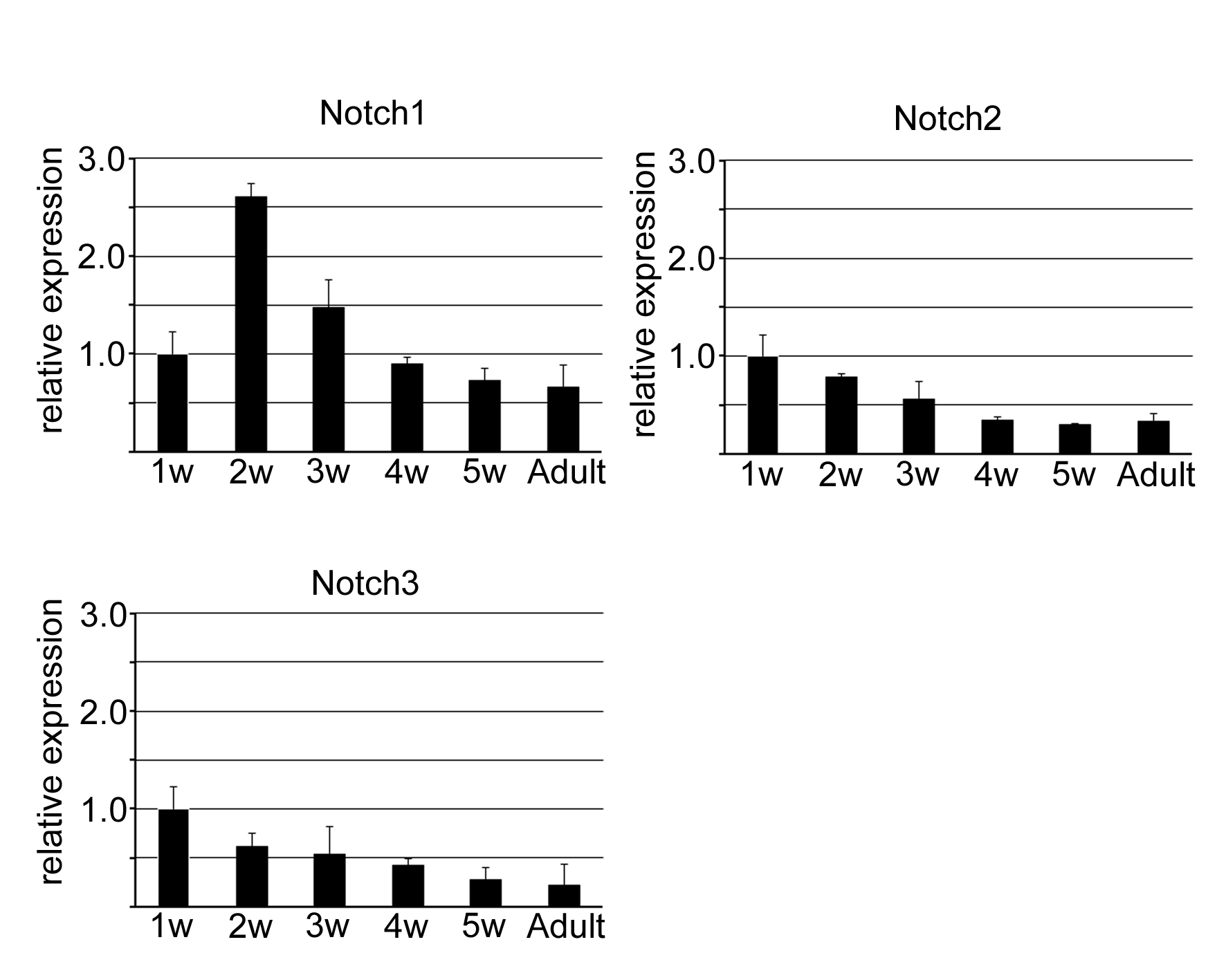

Supplement: S8 Fig — The expressions at 1 week were assumed to equal 1. Error bars indicate standard deviation from the means. *P<0.05. (TIF) [file pone.0124293.s008.tif]
