# Supplementary material for: A Novel Transcriptional Factor Nkapl Is a Germ Cell-Specific Suppressor of Notch Signaling and Is Indispensable for Spermatogenesis
Source: PLoS One. 2015 Apr 14;10(4):e0124293. doi: 10.1371/journal.pone.0124293 (PMC4397068; doi:10.1371/journal.pone.0124293)
Supplement: S1 Table — (DOCX) [file pone.0124293.s009.docx]

| Genotype |  | **+/-** | **-/-** |
| --- | --- | --- | --- |
| Male | Fertility (no. of fertile males/no. of males) | 10/10 | 0/10 |
|  | Litter size (avg. no. of newborn pups) | 6.8±0.6 | 0 |
| Female | Fertility (no. of fertile females/no.of females) | 10/10 | 10/10 |
|  | Litter size (avg. no. of newborn pups) | 6.8±0.9 | 6.9±1.1 |

Values are means ± SEM.
