# Supplementary material for: A Novel Transcriptional Factor Nkapl Is a Germ Cell-Specific Suppressor of Notch Signaling and Is Indispensable for Spermatogenesis
Source: PLoS One. 2015 Apr 14;10(4):e0124293. doi: 10.1371/journal.pone.0124293 (PMC4397068; doi:10.1371/journal.pone.0124293)
Supplement: S2 Table — All values are means ± SEM. Significant differences (P<0.01) are discussed here. (DOC) [file pone.0124293.s010.doc]

|  | | |
| --- | --- | --- |
| Parameter | Value for *Nkapl* ^+/-^mice | Value for *Nkapl* ^-/-^ mice |
| Body weight (N=10) |  |  |
| male | 30.0±2 | 29.8±1.2 |
| female | 26.6±2.3 | 24.6±2.6 |
| Wet weight of organs/ Body weight (N=6) |  |  |
| Testis | 2.01±0.02 | 0.10±0.01 |
| Epididymis | 0.10±0.017 | 0.089±0.005 |
| Seminal vesicle | 0.33±0.04 | 0.29±0.04 |
